# Bracelet- and self-directed observational therapy for control of tuberculosis: study protocol for a cluster randomized controlled trial

**DOI:** 10.1186/s13063-017-1996-2

**Published:** 2017-07-04

**Authors:** Ruixue Huang, Guofeng Ren, Jianan Hu

**Affiliations:** 1 Department of Occupational and Environmental Health, Xiangya School of Public Health, Central South University, Changsha, Hunan Province 410078 China; 2 Department of Nutrition and Food Hygiene, Xiangya School of Public Health, Central South University, Changsha, Hunan Province 410078 China

**Keywords:** Adherence, Low-resource environments, TB

## Abstract

**Background:**

Approximately 80% of global tuberculosis (TB) cases occur in low-resource settings, with little opportunity for TB control. We hypothesized that the rapid increase in smartphone users and advances in digital technology would render bracelet-based applications possible; specifically, that bracelet- and self-directed observational therapy (BSDOT) can be used by patients with TB to ensure adherence to TB medication regimens and by basic village physicians to monitor care. This will ultimately allow TB to be controlled in low-resource environments.

**Methods and design:**

This study will have three phases: development of a bracelet capable of storing pills and recording adherence to medication regimens; creation of a BSDOT smartphone application capable of supporting reminders to patients and health care interactions between patients and village physicians; and performance of a cluster randomized controlled trial in Hunan Province, China. Patients in the intervention group will receive free bracelets and smartphones, and their daily medication intake will be directed by the smartphones; the control group will receive no intervention. The primary outcome will be the TB treatment result as defined by the World Health Organization (WHO) as follows: Cured, Treatment completed, Treatment failed, Died, Lost to follow-up, Not evaluated, or Treatment success. The secondary outcome will be treatment adherence, defined as the percentage of patients receiving TB treatment who missed fewer than 5% of doses. We will also assess self-reported adherence using the Morisky, Green, and Levine Adherence Scale (MGLS) and evaluate respondents’ knowledge about TB and quality of life. A regression model will be used to explore whether the interventions improve drug adherence and other outcome measures.

**Discussion﻿:**

This will be a powerful means by which to strengthen TB control and prevent TB, especially multidrug-resistant epidemics of the disease. In addition, our novel smartphone-based tool can be readily adopted for use in low-resource remote environments with limited health care facilities and few economic assets.

**Ethics and dissemination:**

The protocol has been approved by the Ethics Committee of Xiangya School of Public Health, Central South University (reference number: XYGW-2016-14).

**Trial registration:**

Chinese Clinical Trial Registry, ID: ChiCTR-IOR-16008424. Registered on 5 June 2016.

**Electronic supplementary material:**

The online version of this article (doi:10.1186/s13063-017-1996-2) contains supplementary material, which is available to authorized users.

## Background

Tuberculosis (TB) is an infectious disease caused by the bacterium *Mycobacterium tuberculosis* (MTB). Tuberculosis is diagnosed by identifying MTB bacteria in clinical specimens. A complete medical evaluation must include a medical history, a physical examination, a chest X-ray, and microbiological examination of sputum or another appropriate sample. The disease that has compromised health for 1000 years is one of the greatest global public health challenges of our time [1, 2]. TB is among the “top 10” causes of mortality from disease [3, 4]. In 2014, six million cases of TB were reported to the World Health Organization (WHO). Worldwide, almost one third of all affected people are infected with *M. tuberculosis*, the bacterium that causes TB; eight million people develop TB annually and 1.8 million die of the disease [3]. The greatest disease burdens are found in the poorest populations that have the fewest resources to devote to TB control. China has the second largest TB burden worldwide, accounting for 12% of all cases [5, 6]. Lack of medication adherence to TB treatment regimens and inappropriate prescription practices are important contributing factors to treatment failures and the development of multidrug-resistant (MDR) TB [7, 8] which is now a major threat to the progress made in global TB treatment in recent years. In the 2010 National Tuberculosis Prevalence Survey, 20% of TB patients treated by the public health system—using national TB case-management approaches—were lost to follow-up or were not taking their medications regularly [9, 10]. Therefore, the Chinese medical community needs to implement more effective medication adherence strategies. We cannot confirm that China will meet this target [11], especially considering the increasing numbers of newly diagnosed TB cases, particularly of multidrug-resistant tuberculosis (MDR-TB) [12]. MDR-TB is TB caused by bacteria resistant to treatment by at least two of the most powerful first-line anti-TB medications (drugs), oniazid and rifampin. Some forms of TB, termed extensively drug-resistant TB (XDR-TB), are also resistant to second-line medications.

The increasing incidence of MDR-TB (more contagious, more severe, and deadlier than other forms of TB) in China indicates that the road toward TB control remains long. The problem is not confined to China; worldwide, the spread of MDR-TB is a real threat to TB control [13], even in the United States, a developed country. In San Diego, 22% of TB cases reported in 2012 were resistant to at least one of the four major TB drugs; in New York City, the number of MDR-TB cases in 2012 was twice that in 2007 [14]. The WHO reports that China has the highest disease burden worldwide, followed (in order) by India, Russia, and South Africa. To conduct directly observed therapy (DOT) more effectively, we propose implementing bracelet and self-directed observational therapy, designed to improve TB treatment outcomes in low-resource environments.

China has a high burden of MDR-TB; approximately 54,000 (range: 48,000–61,000) pulmonary cases of MDR-TB were reported in 2013 [15, 16]. A key step [17] in TB control is the establishment of a TB database that can be used to develop effective strategies for TB medication adherence [18]. Critically, the effective way toward TB control is compliance with medication regimens; the need to endure long-term treatment remains a severe problem. Many factors compromise compliance with treatment regimens [19]. Poor health knowledge, a lack of understanding of the benefits afforded by treatment, side effects, and cost are major barriers to treatment success [20]. Almost 12% of patients in Jiangsu Province, China failed to take at least 10% of their prescribed doses of anti-TB medications; this was especially common when life resources were limited [21]. A study conducted in Hebei Province revealed that 46% of patients failed to take at least 10% of their doses. Several other Chinese studies have reported low levels of treatment adherence by patients with TB [21, 22]. To prevent treatment interruptions, new approaches are needed to overcome barriers within the social and health service contexts.

The applications of mobile information technology continue to multiply, perhaps affording the opportunity to bridge the gaps described above, and, in so doing, to enhance the means by which TB may be controlled in China. We seek to offer hope to both patients suffering from TB and to public health bodies seeking better TB control. We believe that mobile technologies may improve medication adherence, deliver education, and send behavioral intervention reminders.

### Research hypothesis

We hypothesize that BSDOT, engaging patients with TB and village physicians, would allow patients to self-record their daily medications on a bracelet using a smartphone, and then send the data to the village physicians who could thus remotely monitor and document all medications and store patient records securely in the Cloud.

The study will be conducted in three phases: creation of a bracelet for patients with TB, design of a smartphone application capable of supporting medication adherence and bracelet software capable of recording tablet ingestion, and qualitative evaluation using a cohort of 400 patients with TB from both high- and low-resource areas and both urban and remote settings.

### Aims and objectives

We sought to improve treatment outcome in both drug-susceptible patients and those with MDR-TB.

BSDOT will directly intervene when a patient does not take their medication. We will assess whether BSDOT using a novel pillbox and smartphone application increases the adherence of poor, TB-infected, rural subjects living in mountainous regions to antibacterial drug-based treatment regimens; we will record their impressions and their satisfaction in terms of feedback.

## Methods/design

### Trial design

The study will be conducted in three phases: creation of a bracelet for patients with TB, design of a smartphone application capable of supporting medication adherence and a bracelet holding medication tablets, and performance of a qualitative evaluation of 400 patients with TB from both high- and low-resource and both urban and remote settings.

### Study sites

The study will be conducted in western Hunan Province, China. Our selected sites had reliable electricity supplies and coverage by smartphone networks. We will conduct a cluster 1: 1 randomized trial in this area. This region contains ethnic minorities that lag the rest of the country in terms of development; many subjects live in low-resource settings, and TB is usually treated by traditional herbs, folk prescriptions, or not at all. Because the area is remote, the WHO DOT strategy cannot be effectively delivered. We chose this area by reference to its demographic and socioeconomic structures.

**Fig. 1:**
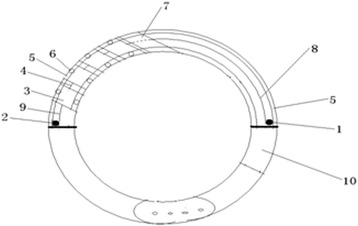
Structure of the bracelet. (1) “Pills-out” traction motor, (2) “Pills-in” traction motor, (3) Pill box, (4) Traction switch, (5) Guide rail, (6) Wheel fixed on the pill box, (7) Display screen, (8) Traction switch between the pills-out traction motor and the pill box, (9) Traction switch between the pills-in traction motor and the pill box

Phase 1: creation of the bracelet: the bracelet is of a polymer composite that is both lightweight and very resistant to damage. The device was able to store pills and to record when pills were taken (Fig. 1).

Phase 2: creation of the BSDOT smartphone application. TB physicians, village physicians, TB managers, nurses, data ethnographers, patients with TB, and medical software designers will form a consensus panel. The application will feature two key modules: a medication adherence record and health care notes. The application will be a downloadable, multiplatform, web-based application, initially for use on mobile phones.

**Fig. 2:**
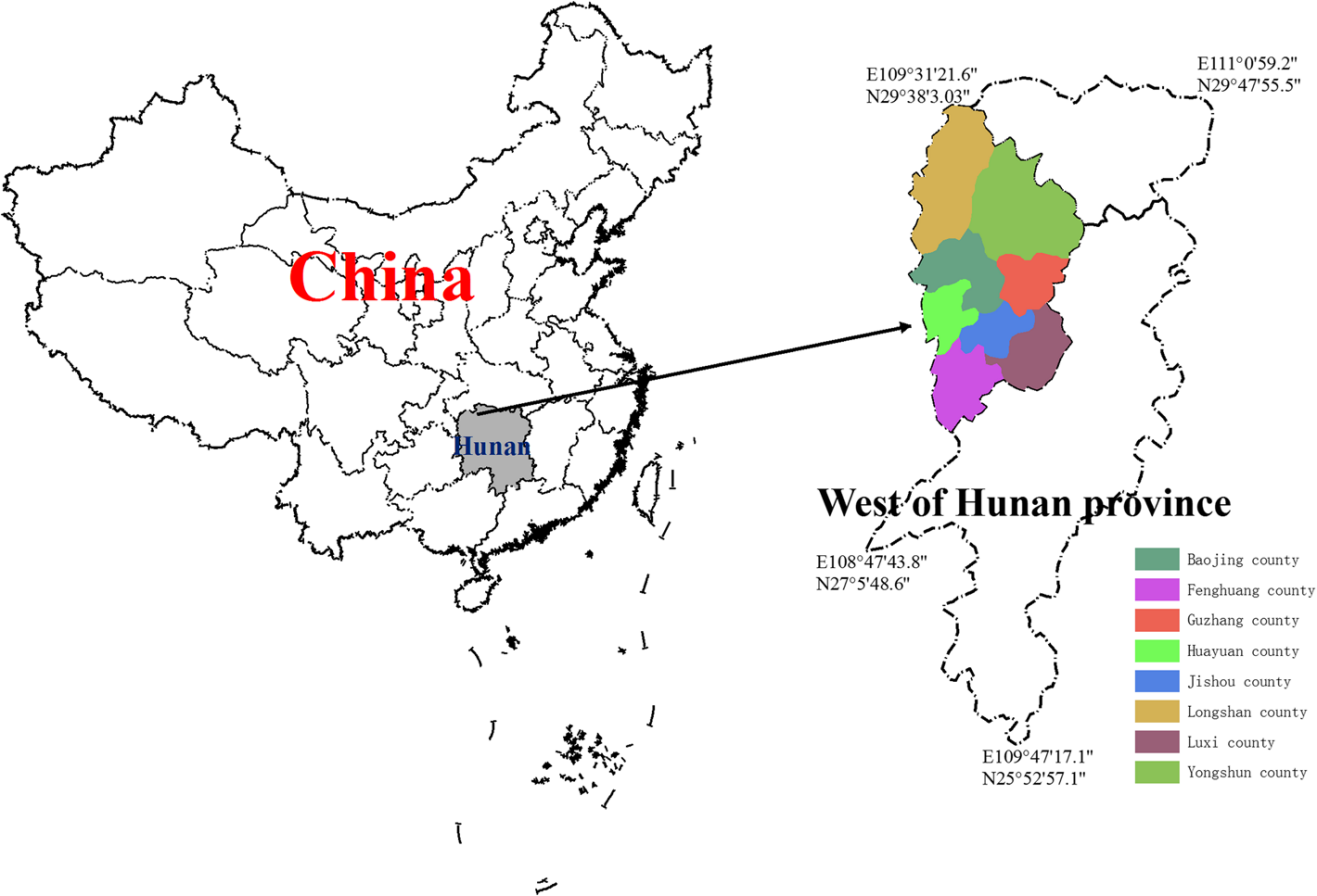
Geographical location of the seven clusters in Hunan Province, China

Phase 3: utility of BSDOT. A 1:1 randomized control trial (RCT) will be conducted among a TB-infected population in Hunan Province with a population of 84.18 million people which is a large agricultural province. The rural population accounts for 63% of the total population of Hunan, one of China’s poorest and most populous provinces. This study is a randomized trial with one intervention group and one control group. For the purposes of this study, counties are defined depending on whether more than half the population are engaged in agriculture and located in rural or mountainous areas. Within Hunan Province, seven counties were selected and assigned a number of TB patients according to the population of each county, such that if the population of the first county was 10% of the total population of the seven counties, we would choose 10% of patients from within this county. Patients selected from each county will be assigned into the control and intervention groups. There is little variation in TB incidence among these seven counties: Luxi county has 150 villages and a population of 273,361; Fenghuang county has 340 villages with a population of 350,195; Huangheng county has 288 villages and a population of 288,082; Baojing county has 314 villages and a population of 277,379; Guzhang county has 140 villages and a population of 126,756; Yongshun county has 327 villages and a population of 428,373; and Longshan county has 462 villages and a population of 384,000 (Fig. 2). All participants will provide written informed consent. The following inclusion criteria will be used: (1) aged 18 years or older, (2) living in villages in west Hunan Province and diagnosed with drug-sensitive pulmonary TB, and (3) possessing the cognitive capacity to understand and follow study procedures and the ability to provide informed consent. The following exclusion criteria will be applied: (1) those who have serious mental or nervous system disorders or with advanced tumors, (2) those who are critically ill, (3) those who have been involved in other studies that assess or may affect treatment adherence, and (4) pregnant women.

**Fig. 3:**
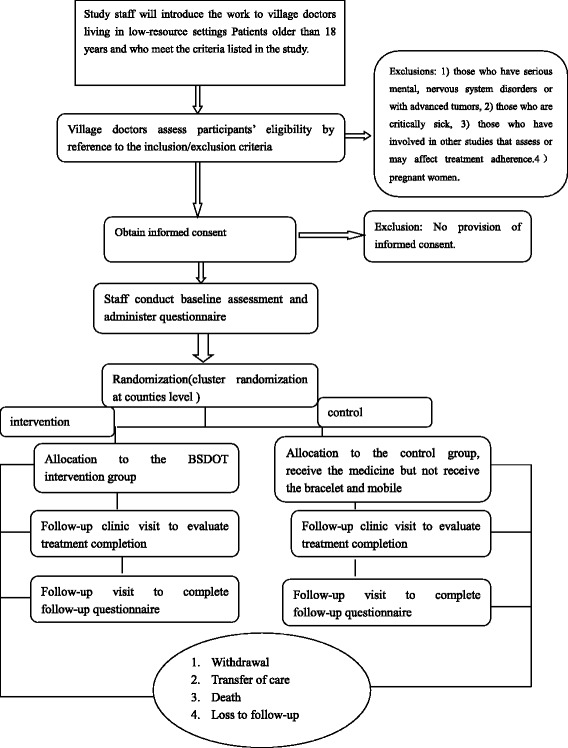
Flow of participants through the bracelet- and self-directed observational therapy (BSDOT) study

**Fig. 4:**
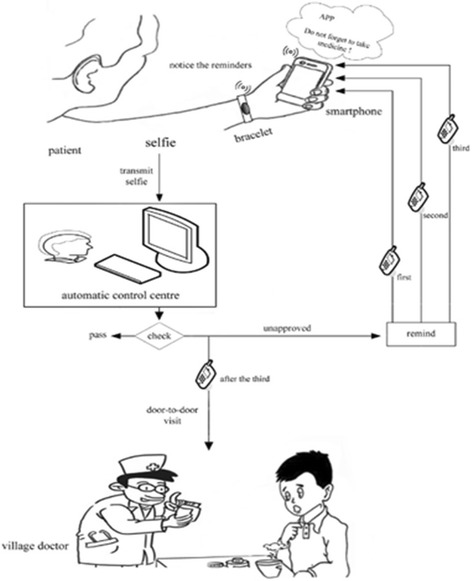
Introduction to the bracelet- and self-directed observational therapy (BSDOT) study

All participants will receive a monthly multidisciplinary check-up and will be followed up for 6 months, during which time adherence will be monitored. Participants in the intervention group will take the medication from an innovative bracelet (Figs. 3 and 4); the bracelet has 14 small compartments in which pills are stored. The bracelet also features two sensors measuring blood pressure and heart rate. The box will open at the time that the patient is scheduled to take the medication. The software combines all the information gathered by the sensors and sends it to the health care system. When a patient takes a pill, they take a self-photograph using the smartphone; this is automatically sent to the monitoring center. If the patient does not send the photograph, the center will automatically remind the patient to take the medication; this reminder will be sent three times. Once the patient replies to the reminder with a photograph, reminders will be stopped for that day. Based on the data available at the center, patients in the intervention group will be divided into three profiles: reminders for timely self-medication, reminders for 2-week follow-up visits, and reminders for the village physicians to switch patients with adherence issues to a more intensive treatment-monitoring method. At each 2-week follow-up visit, the center will evaluate adherence. We will define missed doses as the larger of (1) missed doses based on pill count or (2) missed doses based on a missing photographic reply or a failure to open the bracelet box. If one to two doses are missed in the first 2 weeks, we will instruct the village physicians to talk to the patient about the importance of medication adherence. If three to six doses are missed, we will instruct the village physicians to switch the patient to a more intensive regimen that involves visiting the patient once per week during the study. If seven or more doses are missed, we will switch the patient to the DOT where, according to the policy, the patient will have to take the medication under supervision by the village physicians.

### Assignment to the intervention and control groups

In the intervention group, after signing the Informed Consent Form, each participant will be provided with a bracelet, a battery charger, medication, and a smartphone. The village physicians will insert 2 weeks’ worth of medication into the bracelet, tell participants how to use the bracelet, and explain how to refill the bracelet after 2 weeks. Baseline participant information will be collected via structured questionnaires. Participants will use the bracelets for 6 months. The control group will receive neither the bracelet nor the mobile application; rather, they will receive a traditional TB treatment regimen under the DOT strategy, which includes the same medications as the intervention group. The intervention group’s medication adherence will be assessed using the BDOT strategy, while participants in the control group will visit the physicians for treatment under direct observation. Pill consumption will be assessed by the village physicians during follow-up visits scheduled at 2-week intervals.

### Outcomes and additional measurements

The primary outcome will be the TB treatment result as defined by the WHO, as follows: Cured, Treatment completed, Treatment failed, Died, Lost to follow-up, Not evaluated, or Treatment success (details available at https://www.ncbi.nlm.nih.gov/books/NBK214446/table/annex2.t1/?report). The secondary outcome will be treatment adherence, defined as the percentage of patients receiving TB treatment who omitted less than 5% of their doses. We will also assess self-reported adherence using the Morisky, Green, and Levine Adherence Scale (MGLS), and we will explore whether the respondents know that TB can affect quality of life (QOL). In the control group, the percentages of patients receiving TB treatment who omit fewer than 5% of their doses by the end of treatment, will be determined by the village physicians.

The “knowledge” variable will be assessed by determining whether the patient can correctly answer 10 questions after having been appropriately educated. QOL refers to general wellbeing, including the ability to balance the negative and positive features of life. Life satisfaction includes physical health status, the family situation, educational attainment, employment status, wealth, religious beliefs, the financial situation, and the environment. The EuroQol five dimensions (EQ-5D) module will be used to assess QOL.

### Training assignments for village physicians

Village physicians (“barefoot doctors”) deliver both primary and preventative care to the residents of almost every village in China. In recent years, the Chinese government has promulgated a series of policies mandating upgrades of village clinics, strict clinician training, and remotely delivered education designed to improve the medical knowledge and clinical competence of village physicians. Therefore, we suggest that village physicians have attained the status of qualified health personnel. TB village physicians in both the intervention and the control groups will be trained to understand the study inclusion criteria. When they encounter patients who precisely match the inclusion criteria, they will register such patients and inform the research team. In addition, village physicians and computer clerks in the intervention clusters will be trained in terms of study proposals and procedures. They will understand the study purpose, know how to comply with the proposals, and be able to perform study-related paperwork, including the distribution and collection of weekly Side-effect Report Forms and medication intake self-photographs. Village physicians in the intervention clusters will be trained to provide TB patients with 14-day supplies of TB medications in their bracelets.

### Household-level intervention

Aside from the village-level intervention, additional household-level intervention is very important in the context of the RCT design. Social support may modulate adherence to TB medication protocols; we will thus collect household-level data. Because TB is highly infectious, it is not rare that more than one member of a household will become infected. It is important to be prepared for this situation by collecting relevant data in the initial questionnaire. However, interaction between members of a household will influence the groups’ results. To solve this problem, a great deal of work is necessary to better inform the patients and their families of the treatment regime. Furthermore, following up patients is extremely important. Once a member of the household is infected, chemotherapy treatment is necessary. Finally, it is important to educate the family members on nutrition, exercise, and proper household ventilation.

### Individual-level interventions

Patients in the intervention group will undergo an interactive training course before the research commences. The course will cover bracelet design and application, early symptoms of TB, routes of TB transmission, effects of TB on relatives, TB treatment strategies, the importance of adhering to medication regimens, how to prevent TB, medication side effects, how to open the bracelet, how to switch on the power, how to use a smartphone to download the application, and how to communicate with the village physicians using the application. The course will run for three 90-min sessions. The primary objective will be to encourage patients with TB to take responsibility for their treatment.

After completing the course, the patients will visit their village physicians monthly for medical check-ups and to receive their TB medication for the next month. The village physicians will also check the bracelet power and address any concerns of the patients.

Each day, the patients will photograph themselves taking their medication using a smartphone; these photographs will be automatically sent to the control center, where the photographs will be assessed. TB patients in the interventional group will receive TB-relevant education from the research term, delivered via the smartphones. Patients with TB in the control group (thus, not following the BSDOT strategy) will be treated according to WHO recommendations; a physician must observe the ingestion of TB treatment pills on 6 days per week. Otherwise, patients in the control group will not receive any additional intervention. Research staff will record the following baseline factors: sociodemographic characteristics including age, sex, socioeconomic status, educational level, marital status, and health behaviors (smoking or drinking status). These baseline data will be used to test the accuracy of randomization and to control for confounding effects on primary and secondary outcomes. The patients in the control group also receive TB education, although it generally comes from village physicians or media sources such as newspaper articles or television broadcasts. Pill consumption frequencies by control groups will be assessed by the village physicians during the 2-week follow-up visits. Some drugs may cause adverse effects such as such as vomiting and diarrhea. Any reports of such conditions require careful analysis; it may be possible to adjust the drug dosage or to avoid taking medication on an empty stomach. Also, it may be useful to regularly measure liver and kidney functions. Treatment may be terminated if the adverse effects are very severe. However, prior to study commencement, patient education in terms of drug effects, dose susceptibilities, and possible adverse effects is essential

### Recruitment

The village physicians treating patients in both groups will be trained to collect and report all new and previously treated drug-sensitive cases of TB. Our research staff (who have professional medical knowledge) will be trained on the protocols and procedures of the study and will initially contact eligible patients after (1) village physicians identify patients who meet the study criteria, (2) eligible patients have been contacted by telephone, (3) patient eligibility has been confirmed, (4) the research project has been explained to patients with TB by telephone, patient consent has been gained, and contact information recorded, and (5) interviews have been arranged between patients with TB and other research staff. The research staff will also ask patients about their smartphone usage and the number of smartphones in the household. The average duration of the continuation phase of drug-sensitive TB treatment will be about 6 months.

### Sample size

Sample size calculations were based on a cluster randomized trial; the endpoint was the nonrecovery rate at the end of the trial. Assuming seven clusters per arm, a two-sided type-I error of 5%, a type-II error of 10%, and a percentage with nonadherence in the control arm of 30%, an intra-cluster correlation coefficient (ICC) of 0.1 was estimated as a mid-range value from a list of ICCs from similar studies in terms of intervention targets, outcomes, and units of randomization. Using a treatment effect size of a 0.15 increase in the proportion of successful treatments over the 0.78 successful treatment rate for usual care, based on published local treatment success rates and findings of studies with a smartphone as a facilitator of adherence in similar settings, 1300 TB patients would be required to detect a 40% reduction in the endpoint in the intervention arm with an alpha of 0.05, power of 90%, and coefficient of variation of 0.25. The sample size was adjusted to 1365 assuming 5% missing endpoint data.

### Data collection

To acquire data on secondary outcomes, we will use face-to-face interviews and questionnaires. At baseline (T0) and after completion of TB treatment (T1; follow-up), all patients in both groups will be asked to complete questionnaires.

### Blinding

Participating village physicians and patients with TB cannot be blinded; the BSDOT intervention features open participation. We will blind the research group members involved in data analysis.

### Data management

After the survey is completed, all questionnaires will be checked by one staff member in terms of missing information, unusual responses, and erroneous data. Two staff members will load all data into an SPSS (Statistical Product and Service Solutions, Version 21.0) database; the two databases will then be reconciled to minimize data entry-related errors.

### Statistical analyses

Descriptive statistics will be calculated by reference to baseline data, to allow among-group comparisons. The baseline data will include age, sex, educational level, income, place of birth, smartphone access status, substance use status, distance from home to the nearest county hospital, disease status, and comorbidities. We will compare baseline patient characteristics between the two groups using the chi-squared test or adjusted logistic regression as appropriate. The significance level will be set at *p* ≤ 0.05.

Primary outcomes will be analyzed with the aid of a generalized mixed-effects model featuring both binomial distribution and logistic regression. Randomization (the intervention/control comparison) will serve as an independent variable. To accommodate the clustered design, we will treat clustering as a random effect. Potential confounders (age, sex, socioeconomic status, and other features) differing significantly between the intervention and control groups will be treated as fixed factors. Secondary outcomes (adherence to the medication regime, and education in terms of TB and QOL) will be explored with the aid of generalized linear mixed-effect models employing logistic or linear regression, as appropriate. Secondary outcome modeling will include baseline assessments as covariates. We will report all descriptive and inferential statistics with their 95% confidence intervals [23, 24]. We will treat TB treatment outcomes, treatment adherence, and self-reported adherence as enumerative data. Missing data will be handled by a complete case analysis which excludes individuals with missing data.

### Data safety

Each participant will have a unique identification number. No identifiable information will be included in questionnaires or other data collection forms. All patient information will be kept confidential (under lock and key); electronic patient information will be stored under password-protected access and only the research team will know the passwords. This protects patient privacy. No identifiable information will be provided to persons outside the research team.

### Patient safety and monitoring of adverse events

To minimize potential adverse events, we will utilize bracelet sensors to document daily blood pressure and heart rate in all patients. Moreover, Side-effect Report Forms will be completed daily and reported by telephone to the research team, allowing that team to quickly forward concerns to the TB village physicians for follow-up. Side effects among control patients will be treated when they visit TB outpatient centers.

A Data Monitoring Committee (DMC) will be established by a group of appointed clinicians and biostatisticians who will provide an independent assessment of the safety, scientific validity, and integrity of this study which is expected to have a major impact on clinical practice. The role of the DMC is to provide an added layer of protection for the people enrolled in the study.

Interim analyses will be performed before the scheduled completion of the study. We will consider stopping guidelines based on five possible points: at termination, fixed sample, triangular, double triangular, and restricted procedure. The Standard Protocol Items: Recommendations for Interventional Trials (SPIRIT) 2013 Checklist is available in Additional file 1.

## Discussion

One third of the world’s population is thought to be infected with TB. New infections develop in about 1% of the population each year. In 2014, there were 9.6 million cases of active TB that resulted in 1.5 million deaths. More than 95% of deaths occurred in developing countries. About 80% of people in many Asian and African countries test positive by the tuberculin test; the figure in the United States is 5 to 10%. Any improvement in TB treatment is important in terms of both public health and economics. Improved control and management of TB can lead to better treatment outcomes, thereby decreasing health inequalities, morbidity, mortality, and the economic costs associated with the disease. However, there are several difficulties with the proposed study design. Complications in this project include the developmental costs, the schedule, and the quality of, and the period needed to become used to the wearing of, a bracelet. Another difficulty is widely implementing BSDOT for TB treatment in these low-resource environments as the current administrative management systems in these areas remains underdeveloped. Use of the bracelet seeks to improve medication adherence among TB patients. However, as the technology will be used in low-resource settings, several constraints, including patient educational and income levels and their capacities to treat and control TB, must be relaxed. In terms of access to education, we will train village physicians to understand the program goals, to operate the devices, and to be more familiar with the pathology and clinical features of TB. Thus, the village physicians will be able to tell participants how to use their devices. The low average income of patients constitutes another critical constraint; we will seek to limit the cost of each device to below US$20. The National Bureau of Statistics of the People’s Republic of China (http://data.stats.gov.cn/easyquery.htm?cn=C01&zb=A0A0A&sj=2014) reported that per-capita disposable income of Chinese citizens was US$1613 in 2014. Thus, the device cost would seem reasonable. The economic status of TB patients declines rapidly over time because of the cost of their hospitalization and associated expenses as well as the management of side effects. If adherence to a TB medication plan can be improved, the social cost savings will be much greater than the cost of treatment. It will also be necessary to aim to reduce the cost of the device used to improve medication adherence. Directly observed therapy (DOT) has effectively controlled TB epidemics in China, and is freely available to infected individuals. Thus, if the smartphone application improves medication adherence, we would ask the government to provide free smartphones to TB patients living in low-resource environments. This would reduce device costs. Another constraint is that TB is difficult to both treat and control. Our study region contains ethnic minorities of minimally developed societies. Many patients treat TB using traditional herbs or folk prescriptions, or do not treat TB at all. Such vulnerable populations require special attention.

If our BSDOT strategy is successful, it can be implemented in remote areas (villages or mountainous regions) to improve the quality of overall health care and to ensure better TB control.

### Budget and financial resources

The budget for this study will include the following items: transportation: US$600; bracelet and application costs: US$2000; information costs: US$1000; and experimental charges: US$5000. The total amount comes to US$8600. Financial support for this study will be provided by the Hunan Province Health and Family Planning Commission (BZ2016085).

### Cost-effectiveness of the intervention

Addressing whether the program is cost-effective is also an important consideration for the trial. Our cost-utility analysis will assess the cost per quality-adjusted life-years (QALYs) that were added to the patient’s life as a result of the program. This analysis takes a societal perspective, such that any cost to any stakeholder is accounted for including volunteer time. The lower the cost per QALY is, the more cost-effective the program becomes. In the present study, costs of TB treatment, and those of control of relevant symptoms (e.g., village physicians’ time and medications), will be weighed against costs not incurred (those of hospitalization and the social costs incurred by the disruption of village life). The costs and savings afforded by the proposed study can be compared to those of other medical interventions. We will estimate costs using a Markov chain approach.

### Trial status

At the time of submission of this manuscript, the trial had not begun; enrollment began in December 2016. This project is a first step in improving TB medication adherence in low-resource environments. After the project is completed, we will report the results and conclusions to the Hunan Province Health and Family Planning Commission and submit the findings for publication in an academic journal. We are hopeful that this study will showcase the public health benefits and cost-effectiveness of employing bracelet and application systems for managing TB, which may in turn facilitate policy changes to create systemic incentives to promote DOT medication adherence.

## Limitations

There are several very important limitations in this study that should be mentioned. Firstly, and most obvious, the use of this approach will require access to a smartphone which also requires electricity, access to the Internet, and cell phone reception in the treatment area. Secondly, in rural areas, the WHO DOT treatment may be complicated by the availability of resources; thus, this would render this intervention useless in such settings.

## Additional file

Additional file 1: SPIRIT 2013 Checklist. (DOC 121 kb)

## Abbreviations

BSDOT: Bracelet- and self-directed observational therapy
DOT: Directly observed therapy
DR: Drug resistant
MDR-TB: Multidrug resistant TB
RCT: Randomized controlled trial
SPSS: Statistical Product and Service Solutions
TB: Tuberculosis
WHO: World Health Organization
XDR: Extensively drug-resistant
